# Prevalence of asymptomatic urinary tract infections in morbidly obese dogs

**DOI:** 10.7717/peerj.1711

**Published:** 2016-03-14

**Authors:** Susan G. Wynn, Angela L. Witzel, Joseph W. Bartges, Tamberlyn S. Moyers, Claudia A. Kirk

**Affiliations:** 1 BluePearl Georgia Veterinary Specialists, Sandy Springs, GA, USA; 2 Department of Small Animal Clinical Sciences, College of Veterinary Medicine, University of Tennessee, Knoxville, TN, USA; 3 Department of Clinical Sciences, Cornell University, Cornell University Veterinary Specialists, Stamford, CT, USA

**Keywords:** Obesity, Obesity asymptomatic bacteriuria

## Abstract

**Background.** Obesity has reached epidemic proportions in dogs and, as in humans, cost of care has increased due to associated comorbidities. In humans, asymptomatic urinary tract infections (UTI) may be more prevalent in the obese. Asymptomatic bacteriuria (AB) is the term used when UTI are asymptomatic. We hypothesized that morbidly obese dogs are similarly more likely to have asymptomatic bacteriuria than lean, overweight, and moderately obese dogs.

**Methods.** A retrospective study was undertaken to explore a possible association between obesity and asymptomatic bacteriuria. Records from lean, overweight, and obese dogs receiving both a dual energy absorptiometry (DXA) scan and urine culture were included.

**Results.** Six positive urine cultures were identified among 46 dogs fulfilling search criteria. All six positive cultures were found in dogs with body fat percentage of >45%. In dogs with body fat percentage of <45%, there were no positive urine cultures.

**Discussion.** There was an increased prevalence of asymptomatic bacteriuria in the morbidly obese dogs in this study compared to those that were lean, overweight, or moderately obese. Whether antibiotic therapy is necessary in such cases is still being debated, but because asymptomatic bacteriuria may be associated with ascending infections, uroliths, or other complications, the data reported herein support the screening of obese patients for bacteriuria.

## Introduction

As obesity reaches epidemic proportions in humans, the prevalence and severity of obesity in pet dogs also appears to be on the rise (Lund et al., 2006). It is not uncommon to find morbidly obese dogs whose body fat exceeds the upper limit of most body condition scoring (BCS) systems (>45% body fat) (LaFlamme, 1997; Witzel et al., 2014). The connection between obesity and urinary tract infections (UTIs) in humans is controversial, with some studies showing obese patients have higher rates of UTIs while others find no relationship (Semins et al., 2012; Saliba, Barnett-Griness & Rennert, 2013).

Obesity may increase the risk of UTIs through several mechanisms. Firstly, morbidly obese animals are more likely to suffer from arthritis and joint pain, with overall reduced mobility (Marshall et al., 2009). This reluctance to move can cause animals to void their urine less often and give bacteria a better opportunity to adhere to the bladder mucosa. Morbidly obese animals are also more likely to have infections within the skin folds surrounding the tail base and perineal areas. Ascension of bacteria from the perineal, rectal, and genital areas are the most common sources of bacterial UTIs (Olin & Bartges, 2015).

Asymptomatic bacteriuria is a microbiologic diagnosis that has been defined in the human literature as “the isolation of a specified quantitative count of bacteria in an appropriately collected urine specimen obtained from a person without symptoms or signs referable to urinary infection” (Nicolle et al., 2005). This retrospective study in dogs explored a possible association between morbid obesity and asymptomatic bacteriuria.

## Materials and Methods

### Inclusion criteria

Retrospective data from dogs receiving both a dual energy absorptiometry (DXA) scan and urine culture as part of a previous obesity research study (Witzel et al., 2014) were evaluated. Body condition was assessed by the investigator (AW) using a 5-point BCS system. The original research study from which data was collected was approved by the University of Tennessee’s Institutional Animal Care and Use Committee (Approval # 1866-0909). All dogs underwent a complete blood count and urinalysis, as well as serum biochemistry/electrolyte and total thyroxine testing. All DXA scans and urine cultures were collected within 1 week of each other. No dogs in the original study were symptomatic for UTI.

Because metabolic diseases such as hypothyroidism, hyperadrenocorticism, insulinoma and hypopituitarism may predispose to obesity and complicate its treatment, inclusion in the present study required the dogs be free from systemic medical conditions that would increase their risk for UTIs at the time of DXA and culture. One of the dogs included in this dataset had low total T4 levels, but was not symptomatic for hypothyroidism. No dogs had clinical signs of Cushing’s disease, though further screening tests for hyperadrenocorticism were not obtained. No dogs had evidence of skin infection, and none had owner-reported symptoms indicative of a UTI. No dog had clinical evidence of serious lower urinary tract disease.

### Data collection

#### Urine cultures

Any voided urine or catheterized samples were excluded. Cultures were obtained by collecting 2 mL urine via cystocentesis, after the skin was swabbed with alcohol. Skin infections of the abdomen were not reported in any patient, but because of the retrospective nature of this study, it is possible that mild infections were not noted. Urine was aseptically streaked onto 5% sheep blood agar and incubated at 35 °C in the presence of 5% CO_2_. Cultures were observed daily for bacterial growth. Samples with no bacterial growth were considered negative after 5 days of incubation. Cultures were considered positive if they exhibited any colony growth and if they yielded ≥1,000 colony forming units per mL.

#### DXA

Propofol (2–4 mg/kg (0.91–1.8 mg/lb)) was administered through a cephalic catheter, and DXA was performed with a bone densitometer set up for a whole-body scan. Dogs were placed in sternal recumbency on the DXA table; the scan was initiated cranially, with the dog’s nose behind the table line at the head of the table. The dog’s head and spine were aligned on the centerline, and the carpal joints were flexed to create an approximately 90° angle between each foot and the long axis of the forelimb. When possible, the forefeet were positioned caudal to the base of the skull. The scan field included the entire animal. Lean body mass, fat mass, and body fat percentage were calculated with commercially available software (Apex, version 2.3; Hologic Inc., Danbury, CT, USA).

Following DXA, subjects were categorized as lean (body fat (BF) = 15–24%, BCS 2/5, *n* = 2), overweight (BF = 25–34%, BCS 4/5, *n* = 4), obese (BF = 35–44%, BCS 5/5, *n* = 16), or morbidly obese (BF ≥ 45%, BCS > 5/5, *n* = 24). These categorizations were based on BF percentages correlating with the 9- and 5-point BCS systems used frequently in veterinary medicine (Linder & Mueller, 2014).

### Statistical analysis

A Pearson exact two-sided *χ*^2^ test for independence was used to compare the variable of body fat with the presence or absence of a positive urine culture, and to determine if data distribution differed significantly from that expected if BF and positive cultures were independent (*p* = 0.01, *α* = 0.05). The null hypothesis was that BF and presence or absence of positive culture were unrelated. Adjusted standardized residual (ASR) was also performed, and the data were normally distributed.

## Results

Forty-six dogs with BF ranging from 17–63.7% (mean 43.8; SD 7.6) were included (Tables 1 and 2). Because there was no bacteriuria found in lean, overweight, or obese dogs, these groups were pooled together for data analysis. The prevalence of asymptomatic bacteriuria in morbidly obese dogs was 25% (*n* = 6) and had a strong statistical relationship to BF percentage (*p* = 0.007; ASR = 2.7).

**Table 1:** Description of the study population of dogs (*n* = 46). Six dogs had positive urine cultures.

Breed	Age (years)	Sex	Body fat %
Lean			
Boxer	3	M	17
Mixed	11	NM	20.4
Overweight			
Dachshund	9	NM	28.3
Hound mix	4	NM	27.2
Mixed	2	FS	29
French bulldog	5	FS	34.3
Obese			
Mixed	11	FS	35.5
Labrador retriever	8	NM	36.3
Miniature pinscher	6	NM	38.1
Boxer	3	FS	38.1
Golden retriever	1	FS	39.4
Australian shepherd	10	FS	38.2
Labrador retriever	4	FS	39.1
Dachshund	1	FS	39.6
Labrador retriever	8	FS	39.7
Shetland sheepdog	7	FS	40.3
Curly coat retriever	11	MS	40.4
Mixed	4	NM	40.4
Labrador retriever	6	FS	40.5
Jack Russell terrier	7	NM	42.3
Miniature pinscher	11	F	42.7
German shepherd dog	11	NM	43
Morbidly obese			
Mixed	4	NM	45.2
Border collie	5	FS	45.4^a^
Mixed	3	FS	45.6
Chihuahua	6	FS	46.6
Golden retriever	2	NM	46.7
Corgi	7	NM	47.5
Beagle	7	FS	48.2^a^
English mastiff	11	FS	48.2
Staffordshire terrier	2	NM	48.2
Boston terrier	7	FS	48.3
Labrador retriever	9	FS	48.4
Mixed	8	NM	48.6
Doberman pinscher	7	NM	50.6
Doberman pinscher	8	NM	50.9^a^
Golden retriever	6	NM	51
Rottweiler	8	FS	52.6
Australian shepherd	9	FS	53.2
Beagle	11	FS	53.4^a^
Labrador retriever	6	FS	54.2
Dachshund	4	FS	56.4
Shetland sheepdog	10	FS	56.9
Shih tzu	9	FS	56.9
Shih tzu	7	NM	59.3^a^
Jack Russell terrier	9	FS	63.7^a^

**Notes.**

M male NM neutered male FS female spayed F female

a Positive urine culture (bacteriuria).

**Table 2:** Descriptive statistics of the study population of dogs (*n* = 46).

	% Body fat	
	<**45**	≥**45**
*n*	22	24
Age median (range) in years	6.5 (2–11)	7 (1–11)
Sex		
Male	10	9
Female	12	15

Of the six dogs with positive cultures (Table 3), two were neutered males and four were spayed females. Only one had been previously treated with antibiotics, and all were asymptomatic.

**Table 3:** Characteristics of dogs with positive urine cultures (*n* = 6).

Breed	Age (years)	Sex	Body fat %	Culture details	Urinalysis findings
Border collie cross	5	FS	45.4	Beta-hemolytic *Streptococcus*: 15,000 CFU/mL *Staphylococcus pseudintermedius*: 10,000 CFU/mL	USG: 1.020 pH: 7.0 protein: trace RBC: 0.2/HPF WBC: rare
Beagle	7	FS	48.2	*Escherichia coli*: >100,000 CFU/mL	USG: 1.027 pH: 7.0 RBC: rare WBC: TNTC Protein: 1+
Doberman pinscher	8	MN	50.9	Beta-hemolytic *Streptococcus*: >100,000 CFU/mL	USG: 1.007 pH: 7.5 RBC: 0/HPF WBC: 0 Protein: negative
Beagle	11	FS	53.4	*Enterococcus* NOS: 100,000 CFU/mL	USG: 1.048 pH8.0 RBC: rare WBC: 2–3/HPF Protein: 2+
Shih tzu	7	MN	59.3	*E. coli:* 3,000 CFU/mL gram-negative rods: 2,000 CFU/mL	USG: 1.045 pH: 6.0 RBC: 0–2/HPF WBC: 2–3/HPF Protein: 3+
Jack Russell terrier^a^	9	FS	63.7	*E. coli:* >100,000 CFU/mL	USG: 1.025 pH: 7.0 RBC: rare WBC: 3–5/HPF Protein: 2+

**Notes.**

FS female spayed MN male neutered CFU colony-forming unit USG urine specific gravity RBC red blood cell count WBC white blood cell count HPF high-power field TNTC too numerous to count NOS not otherwise specified

a Dog previously received enrofloxacin antibiotic therapy.

## Discussion

In developed countries, obesity is the most common nutritional disorder in companion animals (German, 2006). Recent studies indicate that 34–59% of dogs presented to veterinary practices in Europe, Australia and the United States are overweight, and 5–20% are obese (McGreevy et al., 2005; Colliard et al., 2006; Lund et al., 2006; Courcier et al., 2010). Obesity in dogs is associated with several comorbidities, including pancreatitis, osteoarthritis, oral disease, neoplasia, heart disease (Thengchaisri et al., 2014), and lower urinary tract disease. Diabetes mellitus, a risk factor for UTIs, is associated with obesity, as well. Urinary tract infections are most common in females and may lead to ascending infections (such as pyelonephritis), urolithiasis, and other complications. A significant proportion of antibiotics used in small animal practice are prescribed for these conditions.

Both BCS and morphometric equations describe only dogs with 40% BF or less. There are no defined terms for overweight, obese, or morbidly obese dogs. In humans, BMI can be used to estimate BF percentage according to the following equation, where sex is 1 for males and 0 for females (Deurenberg, Weststrate & Seidell, 1991):

\begin{eqnarray*}\text{ Adult body fat %}=(1.20\times \mathrm{BMI})+(0.23\times \mathrm{age})-(10.8\times \mathrm{sex})-5.4. \end{eqnarray*}

In humans, a BMI > 40 is considered morbidly obese (Shikora, 2005). We designated dogs with a BF percentage of greater than or equal to 45% as morbidly obese, correlating them to the calculated BF percentage in a 45-year-old male or female and a BMI of >40, which results in a BF approximating 40–50%.

A UTI occurs when normal host defenses fail (Seguin et al., 2003). These defenses include anatomical structures, normal micturition, mucosal defense barriers, anti-bacterial properties of urine itself, and systemic immune competence. Obese patients may experience breaks in these multiple defenses against UTIs. For instance, excess body weight tends to reduce daily activity level in dogs (Manens et al., 2014; Morrison et al., 2013). A reduction in activity may lead to urinary retention due to fewer episodes of micturition daily. As BF accumulates, a change in the anatomy surrounding the urethral orifice may increase bacterial numbers and lead to dermatitis in the area. Obesity may cause a hooded vulva, which is an established cause of UTI recurrence in females.

Mucosal defense barriers include the mucosal immune system, as well as the normal microbiome. The urinary tract has historically been considered sterile, though recent evidence suggests there is a urinary bladder microbiome in addition to the gastrointestinal microbiome (Whiteside et al., 2015). Some of the same species that can cause UTIs may also survive in the urinary tract without causing true infection. Studies in the gastrointestinal tract have shown that the microbiome is critical in the maintenance of health and the development of disease. The canine microbiome remains to be well characterized, but preliminary studies suggest that the microbiome of obese dogs contains less diversity than the microbial community of lean dogs (Handl et al., 2013; Park et al., 2015), which may influence mucosal immunity in this population of dogs. Although UTIs have been thought to occur from infection by gastrointestinal bacteria, an additional role for the gut microbiome may occur secondary to renal filtration and bladder storage of metabolites formed by the gastrointestinal microbiome. Therefore, changes in the gastrointestinal microbiome may have a role in urinary homeostasis (Whiteside et al., 2015). The altered microbiome harbored by morbidly obese dogs may offer one explanation for our results.

The significance of positive urine cultures without overt clinical signs of infection remains unclear. In human medicine, asymptomatic bacteriuria is usually transient and does not require antibiotic therapy (Nicolle et al., 2005). In a study evaluating female dogs with asymptomatic bacteriuria, about half the dogs had transient colonization while the other half had bacteriuria persist for at least 3 months. No dogs in either group developed clinical signs throughout the study (Wan et al., 2014). Despite evidence indicating that asymptomatic bacteriuria is non-progressive in healthy dogs, the morbidly obese population may require additional clinical assessments, such as routine urinalyses. While treatment of asymptomatic UTIs is debatable, veterinarians should be aware that morbidly obese dogs are at higher risk for UTIs and may benefit from routine screening for such infections. The factors placing them at a higher risk for bacteriuria may also prevent them from clearing infections. A constant influx of bacteria or yeast from moist skin folds or a reluctance to properly void bladders may result in numerous or more pathogenic organisms colonizing. Obesity also results in a state of chronic inflammation that may reduce the ability to fight infections (Karlsson & Beck, 2010).

The true risk for asymptomatic bacteriuria leading to a UTI, as well as possibly ascending infections and other complications, remains to be studied. Therefore, controversy exists as to whether asymptomatic bacteriuria should be treated with antibiotics. Extensive medical histories were not available in the retrospective data, and it is unknown whether bacteriuria was new, recurrent, or persistent from previous urinary infections. Additionally, although obesity affects the pharmacokinetics of antimicrobial drugs, no established recommendations exist for antimicrobial dosing in obese patients. The uncertainty regarding appropriate antimicrobial dosing for obese patients may represent yet another risk factor for persistent infections. As antibiotic resistance becomes a more serious concern, preventable infections have come under scrutiny as one way to control cost of care, mitigate antibiotic resistance, and prevent permanent changes in normal microbiomes.

This study had several limitations. Firstly, subjects were recruited for a previous study, and data collection was retrospective. Also, a selection bias may exist due to the subjects being selected specifically for a high BCS, which may be influenced by other comorbidities of obesity.

## Conclusions

This study points out an association between obesity in dogs and asymptomatic bacteriuria. However, these findings cannot suggest cause and effect or be applied to obese dogs as a population. Still, considering the financial costs of treating UTIs, as well as the implications for the emergence of resistant bacteria, further study to determine whether weight loss translates to reduced risk of infection is recommended. The increased prevalence of asymptomatic bacteriuria in the morbidly obese dogs in this study suggests that this population may be useful for further investigation of this question.

## Supplemental Information

10.7717/peerj.1711/supp-1 Supplemental Information 1 UTI obesity full dataset Subject signalment, BCS, and urinalysis findings.

## Additional Information and Declarations

### Data Availability

The following information was supplied regarding data availability: The research in this article did not generate any raw data.
